# PGen: large-scale genomic variations analysis workflow and browser in SoyKB

**DOI:** 10.1186/s12859-016-1227-y

**Published:** 2016-10-06

**Authors:** Yang Liu, Saad M. Khan, Juexin Wang, Mats Rynge, Yuanxun Zhang, Shuai Zeng, Shiyuan Chen, Joao V. Maldonado dos Santos, Babu Valliyodan, Prasad P. Calyam, Nirav Merchant, Henry T. Nguyen, Dong Xu, Trupti Joshi

**Affiliations:** 1 Informatics Institute, University of Missouri, Columbia, MO USA; 2 Christopher S. Bond Life Sciences Center, University of Missouri, Columbia, MO USA; 3 Department of Computer Science, University of Missouri, Columbia, MO USA; 4 Information Sciences Institute, University of Southern California, Los Angeles, CA USA; 5 Division of Plant Sciences, University of Missouri, Columbia, MO USA; 6 National Center of Soybean Biotechnology, Columbia, MO USA; 7 iPlant Collaborative, University of Arizona, Tucson, AZ USA; 8 Department of Molecular Microbiology and Immunology and Office of Research, School of Medicine, University of Missouri, Columbia, MO USA

## Abstract

**Background:**

With the advances in next-generation sequencing (NGS) technology and significant reductions in sequencing costs, it is now possible to sequence large collections of germplasm in crops for detecting genome-scale genetic variations and to apply the knowledge towards improvements in traits. To efficiently facilitate large-scale NGS resequencing data analysis of genomic variations, we have developed “PGen”, an integrated and optimized workflow using the Extreme Science and Engineering Discovery Environment (XSEDE) high-performance computing (HPC) virtual system, iPlant cloud data storage resources and Pegasus workflow management system (Pegasus-WMS). The workflow allows users to identify single nucleotide polymorphisms (SNPs) and insertion-deletions (indels), perform SNP annotations and conduct copy number variation analyses on multiple resequencing datasets in a user-friendly and seamless way.

**Results:**

We have developed both a Linux version in GitHub (https://github.com/pegasus-isi/PGen-GenomicVariations-Workflow) and a web-based implementation of the PGen workflow integrated within the Soybean Knowledge Base (SoyKB), (http://soykb.org/Pegasus/index.php). Using PGen, we identified 10,218,140 single-nucleotide polymorphisms (SNPs) and 1,398,982 indels from analysis of 106 soybean lines sequenced at 15X coverage. 297,245 non-synonymous SNPs and 3330 copy number variation (CNV) regions were identified from this analysis. SNPs identified using PGen from additional soybean resequencing projects adding to 500+ soybean germplasm lines in total have been integrated. These SNPs are being utilized for trait improvement using genotype to phenotype prediction approaches developed in-house. In order to browse and access NGS data easily, we have also developed an NGS resequencing data browser (http://soykb.org/NGS_Resequence/NGS_index.php) within SoyKB to provide easy access to SNP and downstream analysis results for soybean researchers.

**Conclusion:**

PGen workflow has been optimized for the most efficient analysis of soybean data using thorough testing and validation. This research serves as an example of best practices for development of genomics data analysis workflows by integrating remote HPC resources and efficient data management with ease of use for biological users. PGen workflow can also be easily customized for analysis of data in other species.

**Electronic supplementary material:**

The online version of this article (doi:10.1186/s12859-016-1227-y) contains supplementary material, which is available to authorized users.

## Introduction

In-depth informatics analysis of genotypic data can provide a better understanding of genotype-phenotype correlations with applications designed to assist in the work toward improvement of traits. In order to achieve this, many research institutions are generating large-scale sequencing datasets for crop germplasm [1, 2] for a comprehensive overview of the sequence variation observed in these large collections of crops. With the decreasing costs of NGS, many projects can easily generate single and paired end Illumina reads for hundreds to thousands of samples in a short time. These genomics datasets are large and require significant computing time for analysis. SNP/Indel identification procedures need to be followed by other complex downstream analyses ranging from SNP annotations, copy number variations (CNV), genome wide associations studies (GWAS) analysis, haplotype analysis and others. Most analyses need to be conducted on the entire datasets and often need to combine multiple datasets. Not many biological labs generating the data are equipped with large data storage, computing resources or computing skills for handling such analyses in a time sensitive fashion. These analyses take anywhere from a few days to several months, given the volume of NGS samples and datasets sequenced. In addition, many research institutions may not have access to enough dedicated resources available locally to conduct this type of analysis and usually need to work closely with informatics or computational biology collaborators to build such a capacity and tap into the latest emerging computational techniques. There is a significant need for fast, efficient and easy-to-use computational pipelines to be made available to biological researchers, that use the most advanced techniques such as high-performance computing (HPC), cloud storage resources and provide access to remote computing resources with a scalability to meet the demands of such research projects.

Soybean is an important economic crop and is no exception to the computational barriers associated with a lack of access to advanced HPC and other NGS resources just mentioned. Soybean is a great source of dietary protein and oil for human and animal consumption. The soybean community has invested a great deal of efforts in both sequencing germplasm and creating phenotypic datasets, which has resulted in hundreds of resequencing datasets for both cultivated (*G. Max*) and wild soybean genomes (*G. Soja*) [3]. Here we describe our recent informatics workflow and tool development, and its application to NGS datasets in soybean. To analyze these data, we developed “PGen,” a genomic variation analysis workflow using Burrows-Wheeler Aligner (BWA) [4] for alignment and the Genome Analysis Toolkit (GATK) [5] for SNP and indels identification. This workflow can be run in both Linux systems using repository from a GitHub and Pegasus [6] environment, and online via submission through the SoyKB website [7, 8]. We have applied this workflow to analyze resequencing data of a total of 500+ soybean germplasm lines for SNPs and indels calling from multiple datasets. All the soybean results are integrated and available for browsing via SoyKB’s new NGS resequencing data browser available at http://soykb.org/NGS_Resequence/NGS_index.php. PGen workflow can also be utilized for other organisms and crops by easy customization and serves as a good template for reproducible workflow for bioinformatics analysis with different types of NGS data.

## Methods

### Soybean germplasm NGS datasets

500+ soybean germplasm lines were sequenced at different coverages (15X and 40X) from multiple datasets and utilized for SNP and indel identification. Table 1 shows the details for the number of sequenced lines and sizes of raw datasets generated. Each soybean line was sequenced in paired-end 100–150 bp reads using Illumina HiSeq. This generated 8+ TB of raw NGS sequencing reads data for all the projects in total, which needed to be combined and analyzed together for variation detection.

**Table 1:** Details of soybean NGS resequencing datasets generated

Datasets	Number of sequenced lines	Coverage	Raw Data Size (TB)	# of reads (Millions)	Data Source
MSMC	106	14.7	1.9	196.4	Valliyodan et al. 2016 [3]
USB Phase I	300	17.6	3.63	194.40	Unpublished
USB Phase II	50	44.2	1.97	486.24	Unpublished
Soja lines	45	16.7	0.55	182.71	Unpublished
Brazilian lines	28	14.8	0.34	184.87	Maldonado et al. 2016 [22]

### Genomic variations identification with PGEN workflow

**Fig. 1:**
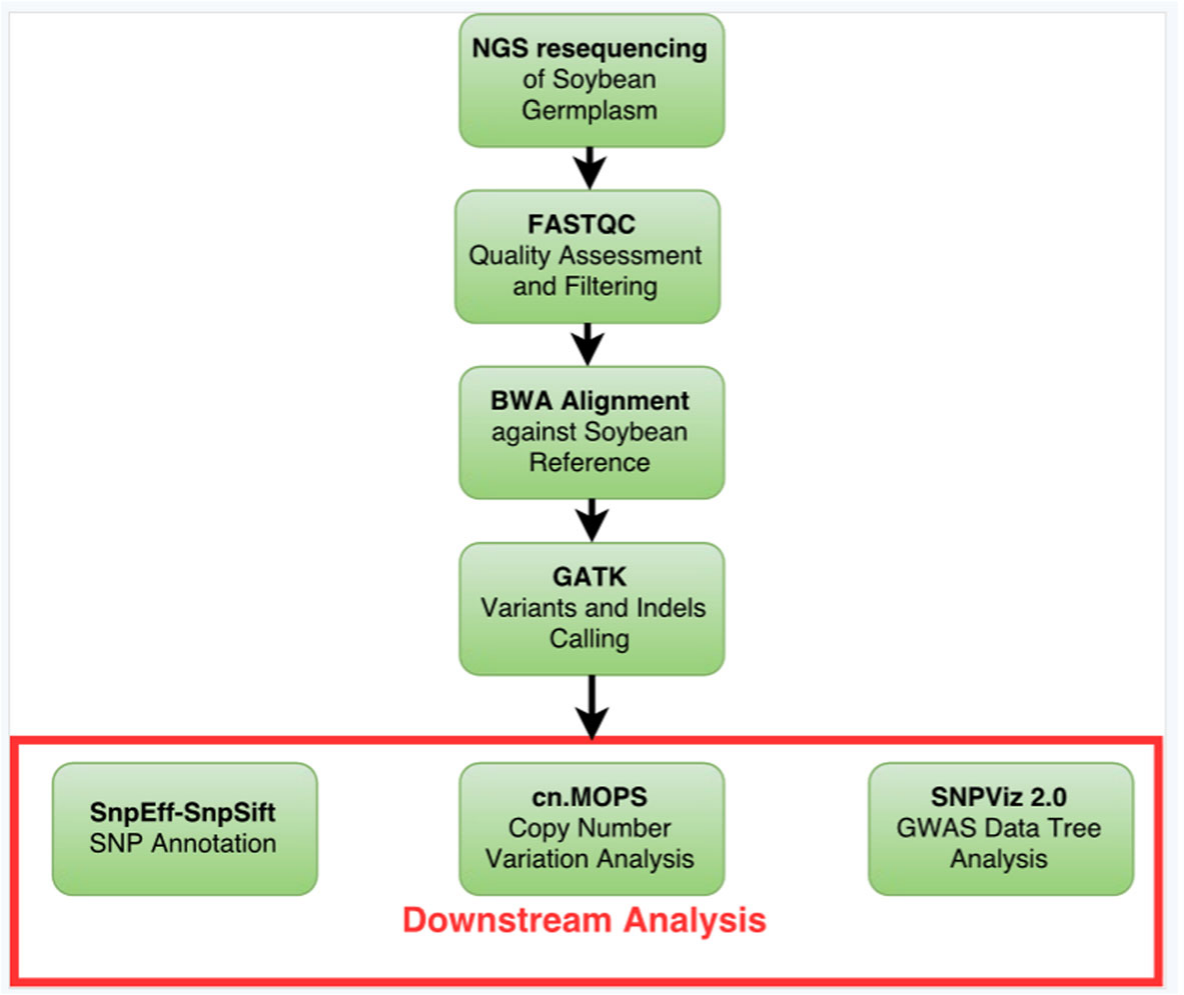
Flowchart of steps and tools utilized in PGen workflow and downstream analysis

*G. Max Williams 82* Wm82.a2.v1 version [9] available via Phytozome [10] was used as the reference genome for this analysis. We built the PGen multi-step workflow using the Pegasus [6] workflow management system (Pegasus-WMS) as shown in Fig. 1, using many widely accepted open-source NGS tools for quality checks, alignment of reads, variants calling, variants filtration, vcf merging and others. The workflow starts by accepting both paired-end or single-end fastq reads from Illumina as input and performs data quality checks as the first step using FastQC [11]. Only the filtered high-quality reads are later aligned against the reference genome using BWA [4]. Picard Tools [12] is also used at this step to locate duplicate molecules and assign all reads into groups with the default parameters. After alignment, SNPs and indels were called using the Haplotype caller algorithm from the Genome Analysis Toolkit (GATK) [5]. Filtering criteria were defined in INFO field in vcf file, where QD stands for quality by depth, FS is Fisher strand values and MQ is mapping quality of variants. Detected variants were then filtered using the criteria “QD < 26.0 || FS > 60.0 || MQ < 40.0” for SNPs and “QD < 26.0 || FS > 200.0 || MQ < 40.0” for indels. Additional filtering can also be applied by modifying the configuration file of a PGen workflow. Outputs are generated as BAM and VCF standard formats that are accessible through both the iPlant data store (iDS) [13] and the SoyKB database via the newly built NGS resequencing data browser. The BAM and VCF results files are further utilized for downstream analyses of SNP annotations, CNVs, and genotype-phenotype relationships. Filtered SNPs and indels are then annotated using SnpEff 3.0 [14]. CNVs among different sample groups are predicted using cn.MOPs [15]. The SNP and indel datasets are directly loaded from iDS into the SoyKB NGS resequencing data browser for visualizing the datasets. In SoyKB the data is also loaded into our SNPViz 2.0 [16] tool, developed in-house for phylogeny analysis using SNPs in selected regions by users.

### PGen workflow optimization using TACC computing resource

The PGen workflow has been optimized to obtain the most efficient and large-scale analysis of the sequenced lines using iDS for cloud storage, Extreme Science and Engineering Discovery Environment (XSEDE) [17]. XSEDE is a high-performance computing infrastructure and Pegasus workflow management system, which we use to control and coordinate data management and computational tasks. iDS was used as the cloud storage infrastructure, and all raw data and final results were stored and managed within it. The Pegasus [6] workflow system is used to define and control the required computational tasks. These include user-defined tasks, such as BWA, Picard, and GATK as well as Pegasus-added tasks such as data staging between the iDS and Stampede/Wrangler’s flash-based file system. Pegasus also adds data cleanup tasks to maintain and minimize the workflow footprint on the file system as the workflow progresses. All computing tasks were performed on the Texas Advanced Computing Center’s (TACC) [18] large-memory and multi-cores Stampede and Wrangler systems with assigned nodes and memory for each single task. Data management was a major challenge for these computations. The NGS resequencing data (~40 TB) is housed in iDS, which is based on iRODS (integrated Rule -Oriented Data-Management System). Key features utilized for this analysis include the ability to replicate data with computational resources utilizing parallel data transfer capabilities, while maximizing available network bandwidth. We split our workflow into multi-steps and parallel processes to gain the most efficiency. Table 2 shows how the sub-applications from BWA, Picard, and GATK require different configurations in terms of number of cores (basic Linux threads) and memory that they can efficiently utilize. Based on our thorough testing, these requirements vary from a single core with 2 GBs of RAM to 16 cores with 22 GBs of RAM. The Pegasus workflow is fully defined with all the requirements for each individual task.

**Table 2:** The PGen workflow consists of several individual tasks with diverse core and memory requirements, which were assigned based on tools’ applicability of multiple threads and memory cost after testing

Tasks	Base code	Cores (Threads)	Memory (GB)
Indexing of reference genome	BWA/samtools/picard tools	1	4
Alignment to reference genome	BWA	1	21
Sorting sam files	Picard tools	1	21
Removal of PCR duplicates	Picard tools	1	21
Add or replace read groups	Picard tools	1	21
Create realign target	GATK_RealignerTargetCreator	15	20
Realign indels	GATK_IndelRealigner	1	10
Calling variants	GATK_HaplotypeCaller	1	3
Select SNPs and indels	GATK_SelectVariants	14	10
Filtering variants	GATK_VariantFiltration	14	10
Create genotype GVCF	GATK_GenotypeGVCFs	1	10
Merge GVCFs	GATK_CombineGVCFs	1	20
Combine variants	GATK_CombineVariants	1	10

**Fig. 2:**
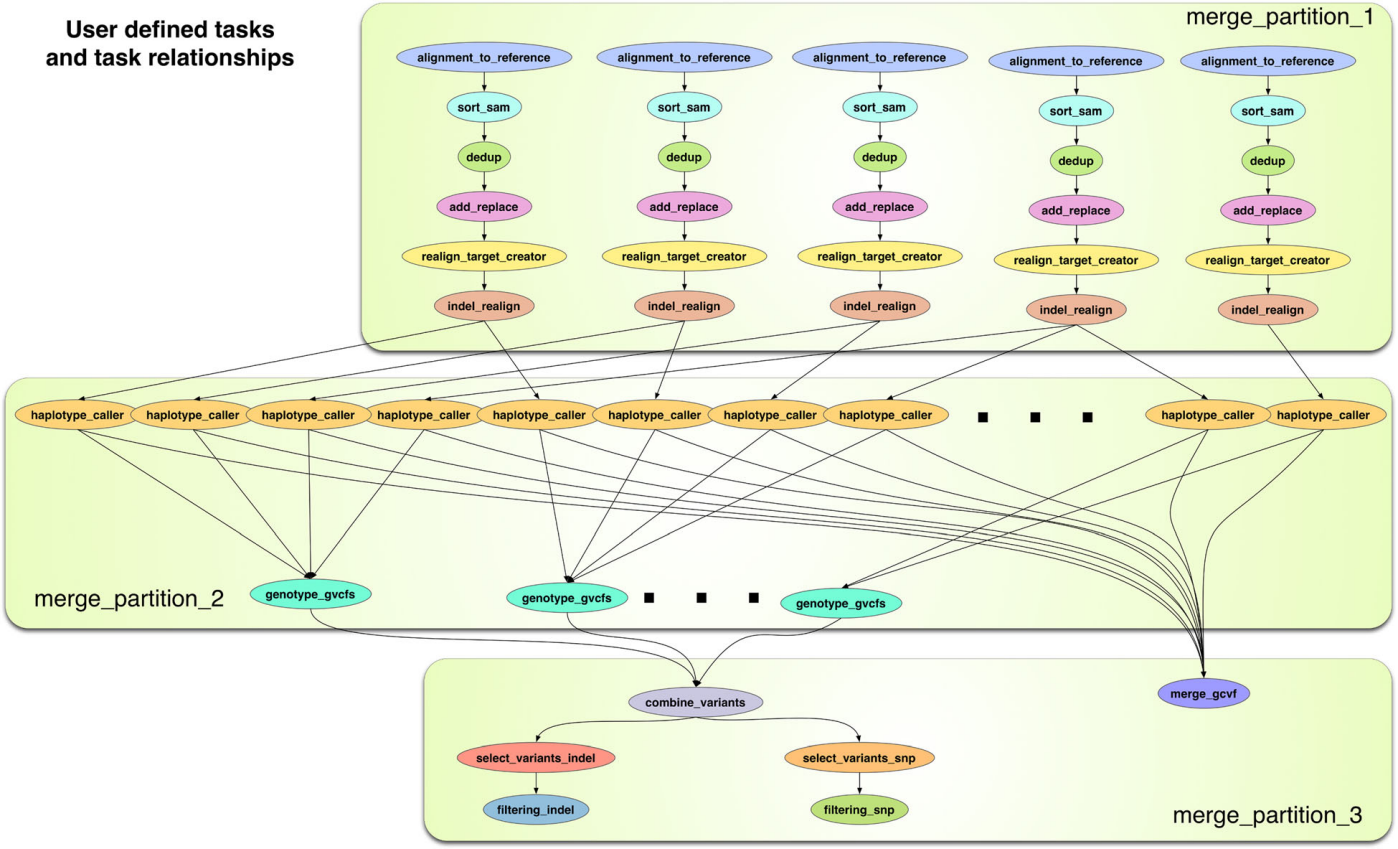
Message passing interface (MPI) jobs within PGen workflow using five soybean line examples

Thorough testing, validation, and optimization were conducted for the PGen workflow. The PGen workflow was split into three message passing interface (MPI) tasks in the workflow as shown in Fig. 2, which gives an example of 5-line analysis. The workflow describes the dependencies among the tasks as a directed acyclic graph (DAG), where the nodes are tasks and the edges denote the task dependencies. These have been created for optimized and fast analysis, such that all individual tasks can complete within the 48 h individual job time constraint for TACC resources. This enables users to submit workflows using *pegasus-run,* keep track of the workflows status using *pegasus-status*, generate statistics using *pegasus-statistics*, identify problems in case of failed workflows using *pegasus-analyzer*, resume the workflows after resolving the problems and remove them using *pegasus-remove*. With this optimization we can conduct analysis on all datasets that have more than hundreds of soybean lines with multiple PGen workflows submitted simultaneously on TACC resources, generating results within a 5- to 10-day timeframe. Each workflow consists of a batch of up to 50 soybean lines. This timeframe includes the associated wait times for individual tasks in queue before they can be executed on Stampede or Wrangler systems. For our datasets, we conducted analyses using multiple workflows submitted simultaneously for a subset of soybean lines, and later combined all results for datasets into the final variant calling using the merged GVCF file in GATK. This ensures that SNP and indel calls were made based on the entire datasets of sequenced lines. Further, the outputs were sent back to iDS and utilized in the downstream analysis as well as loaded into the SoyKB NGS data browser for sharing with biological users. The details for necessary accounts, supported input file formats and output files generated for every workflow are available in PGen documentation (Additional file 1) and on GitHub. We also generated a SNP matrix file in addition to the VCF files for data sharing.

### PGen workflow availability

**Fig. 3:**
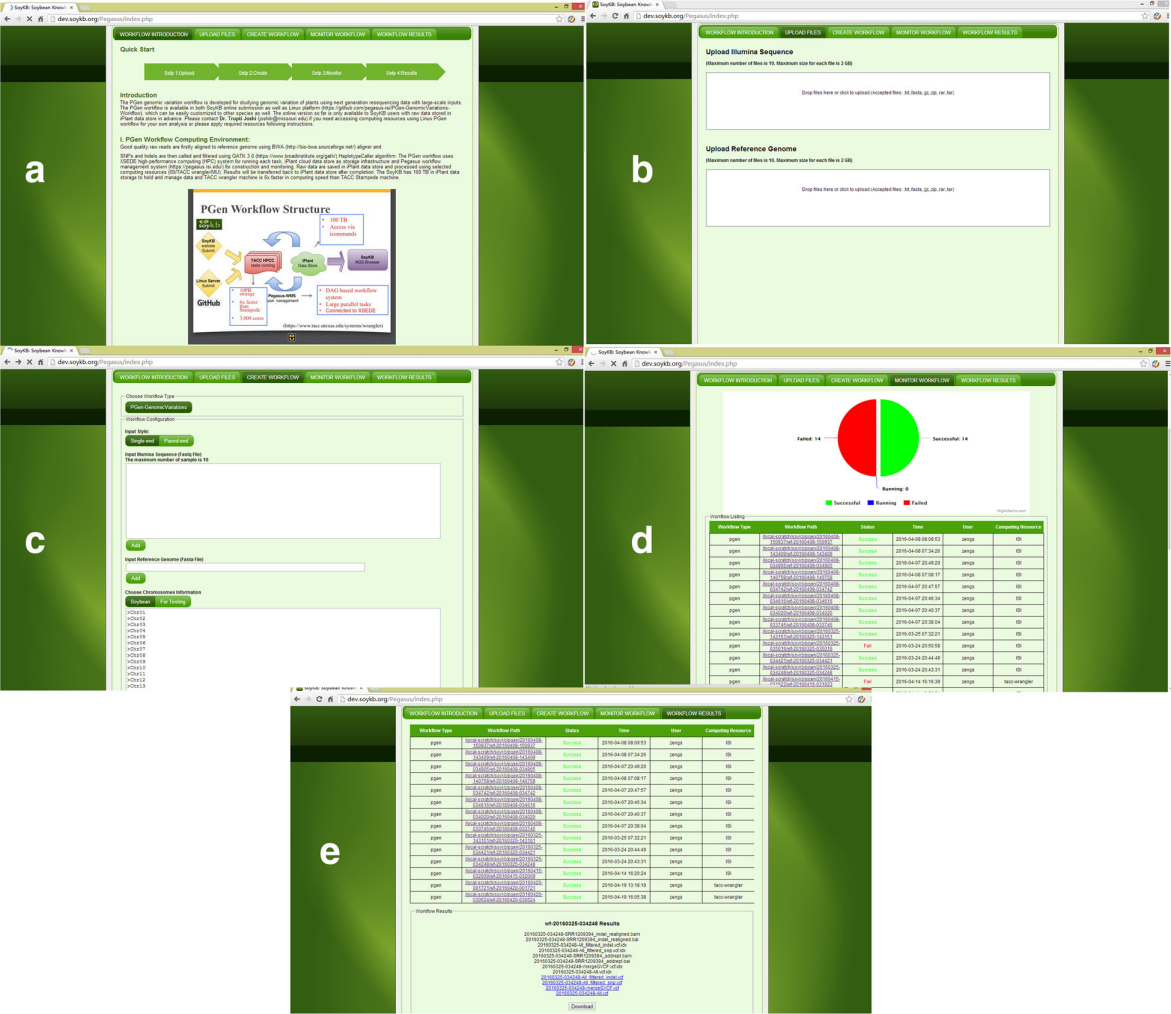
Interfaces of PGen workflow submission via SoyKB website: **a** PGen introduction and structure webpage, **b** upload page for input files to iPlant folder for computation, **c** create workflow page with inputs of raw data, reference genome filtering options and selected computing resource, **d** workflow monitoring with submission history and debug information, and **e** workflow result page for downloading outputs

We made the PGen workflow available in both a Linux version and a web-based implementation integrated within SoyKB [7, 8]. For the Linux version, users can access the workflow via GitHub (https://github.com/pegasus-isi/PGen-GenomicVariations-Workflow) and easily customize it for conducting analysis on organisms other than soybean, uploading reference genome versions of their choice and customizing SNP filtering criteria. For soybean researchers and SoyKB users, the PGen workflow is also available in a web-based implementation within SoyKB (http://soykb.org/Pegasus/index.php) to conduct analysis on their lines of interest and submitting their NGS datasets online. The PGen workflow submission within SoyKB is mainly intended for biology researchers and guides them through five easy steps for workflow creation and submissions, which allows them to access the results within SoyKB as well. The steps for using PGen workflow in SoyKB as outlined in Fig. 3a-e are listed below:

1. *Introduction:* We provide an introduction page, which presents the structure and computing environment of the PGen workflow. A user manual and public data for testing are also provided (Fig. 3a).
2. *Upload data:* The upload data instructional page allows users to upload raw data and reference genome on local machine to SoyKB server and then upload to iPlant data store (iDS) using FUSE mount. Successfully uploaded data will be shown on the create workflow page when selecting inputs (Fig. 3b).
3. *Create Workflow:* The create workflow page connects SoyKB users to the SoyKB data folder on the iPlant and allows them to select raw read fastq files and reference genome fasta file from there as inputs. A workflow is then created using selected variants filtering criteria and computing resources, and a working directory is created for output in the workflow-monitoring page (Fig. 3c).
4. *Monitor Workflow:* Users must be trained to use the PGen workflow history and working directory lists as shown on the workflow-monitoring page. These are used to check the status of workflows, which are shown in pie charts and log histories, which are saved to track error messages for any failed workflows. A statistical summary of computing resources utilized for tasks is generated for all successful workflows (Fig. 3d). Users must learn to use this functionality, which is enabled by linking the PGen workflow in SoyKB with the Narada Metrics system [19]. Sharing statistics and workflow monitoring information is done in real time via the developed RESTful (representational state transfer) APIs (application program interface). Narada Metrics is a software-defined measurement and performance monitoring framework. The framework consists of a Central Intelligence System (CIS) and a number of Measurement Point Appliances (MPA). MPAs are run in a remote distributed resource (such as TACC, Informatics Science Institute (ISI)), which are controlled by CIS to execute workflow on these remote resources, monitor workflow status, collect performance data and send back to CIS. CIS is web service, which provide UI interfaces for users to schedule workflows and view their workflow status.
5. *Workflow Results:* Users can view and download BAM and VCF files of final results as outputs for further merging and conducting downstream analysis when they access the workflow result page (Fig. 3e).

### SoyKB NGS resequencing data browser

We have also developed an NGS resequencing data browser, which is a new suite of tools in SoyKB for the sharing of PGen workflow analysis with soybean researchers. The tool was mainly developed using PHP, Java and JavaScript. This new SoyKB working suite loads data, and provides the results of the workflow and downstream analysis into SoyKB for browsing. We use FUSE to directly mount the iDS folders on the SoyKB servers and avoid replication of huge datasets and associated files. The browser directly retrieves results and displays data in the form of tables using JSON, figures for data quality, and charts for copy number variations, etc.

## Results

### Genomic variations for soybean germplasm lines

We have generated genomics variations and downstream results for all 500+ resequencing lines from multiple projects. On average, more than 99 % of raw reads were mapped to the soybean reference genome. Table 3 below describes a summary of the number of SNPs and Indels, their annotations and CNV results using the PGen workflow. In total, 10,218,141 single nucleotide polymorphisms (SNPs) and 1,398,982 indels were identified. 2,991,576 SNPs were identified in the gene regions, among which 297,254 non-synonymous and 220,927 synonymous SNPs were annotated involving 79,553 soybean transcripts. We also detected 3330 CNVs among these 106 germplasm lines in the MSMC dataset. All SNPs identified by PGen workflow also cover 84.31 % of SNPs from the SoySNP50K [20] SNP array dataset. Identified variants were then used in studies of GWAS analysis using our in-house developed method BHIT [21], and soybean transporter database (SoyTD) analysis. We also analyzed another dataset, which contained 28 Brazilian soybean cultivars sequenced at a coverage of 15X [22]. A total of 5,835,185 SNPs and 1,329,844 indels were identified. 541,762 SNPs, 98,922 indels and 1093 CNVs were identified exclusively in the 28 Brazilian cultivars. Table 4 shows the total CPU running time of jobs and invocations, as well as cumulative job wall time of one maize sample on these computing resources. The results are also available for browsing by soybean researchers via SoyKB.

**Table 3:** Summary of results for NGS resequencing datasets analyzed with the PGen workflow

Datasets	# of sequenced lines	# of SNPs	# of Indels	# of Non synonymous SNPs	# of CNVs
MSMC	106	10,218,141	10,218,141	297,245	3,330
USB Phase I	300	11,972,497	1,590,729	221,013	7,444
USB Phase II	50	7,865,994	1,213,795	152,171	6,892
Soja lines	45	18,066,361	2,198,125	356,129	6,022
Brazilian lines	28	5,835,185	1,329,844	541,762	3,880

**Table 4:** Comparison of running time of PGen workflow of one sample using different computing resources

Resources	Job-runtime (sec)	Invocation-runtime (sec)	Cumulative job wall time	Host
ISI	42374.0	41461.091	8 h, 29 mins	workflow.isi.edu
TACC-Stampede	14054.0	31173.54	9 h, 11 mins	stampede.tacc.utexas.edu
TACC-Wrangler	27146.0	27670.924	3 h, 25 mins	wrangler.tacc.utexas.edu

### SoyKB NGS resequencing data browser

**Fig. 4:**
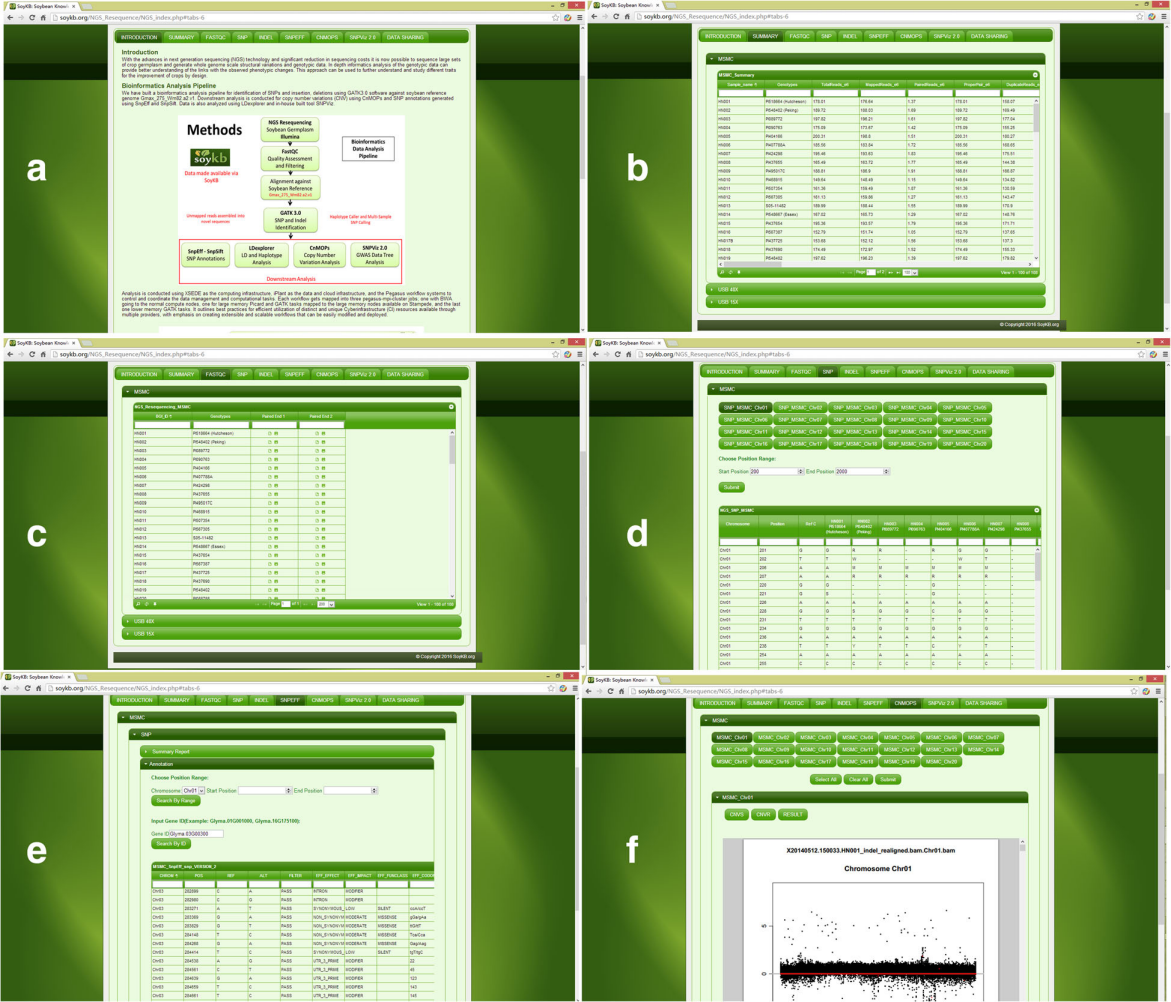
NGS Resequencing Data Browser tool in SoyKB: **a** SoyKB NGS browser introduction and data resource webpage, **b** summary page of sample name, plant introduction (PI) number and raw data information, **c** data quality summary page with downloadable FastQC html report. **d** SNP page of variants information with selected gene region for each soybean line, **e** SNPEFF page showing annotated variant effects for selected gene region or gene IDs, and **f** CNV page with gain and loss CNV region information per chromosome

Our SoyKB NGS resequencing data browser provides an easy-to-use suite of tools for browsing the results of the PGen workflow including the downstream SNP annotations and CNV analysis as shown in Fig. 4. The browser supports each search by gene name, chromosome, start and end coordinates and PI name for germplasm lines. The results can also be downloaded in CSV and PDF format for various analyses. All the tables are searchable and sortable using the text field at the top and clicking on the column names. The NGS data browser provides six different tabs for browsing various types of analysis results generated by PGen as described below:

1. *Introduction:* The introduction tab provides details of different soybean datasets generated from multiple resources. We have analyzed more than 500+ soybean lines using the PGen workflow (Fig. 4a).
2. *Summary:* The summary tab contains plant genotype information (PI name) of sequenced soybean lines as well as statistics related to raw datasets. It provides the total number of raw reads, mapping rates, SNPs and indels identified (Fig. 4b) and other details for every germplasm line.
3. *FastQC:* The FastQC tab provides users access to the data quality results for every line that was generated using FastQC (Fig. 4c). Reports are available for both browsing in a webpage as well as downloading as a zipped file.
4. *SNP:* The SNP tab provides access to the list of filtered SNPs from all analyzed soybean datasets. This tab allows users to search SNPs by selecting a chromosome and entering the start and end coordinates for the region of interest (Fig. 4d).
5. *Indel:* The Indel tab provides access to the list of filtered indels from all analyzed soybean datasets. Indels can also be searched by using a chromosome and coordinates for the region of interest, similar to the SNPs search.
6. *SnpEff Annotation:* The SnpEff tab provides users access to the SNP annotation results computed on the filtered SNPs and indel results using the SnpEff tool. The annotation page displays variant regions on the chromosome, synonymous/non-synonymous effects, amino acid changes, and transcript gene names along with access to the SnpEff html summary page (Fig. 4e).
7. *CNMOPS:* The CNMOPS tab contains results of the CNV analysis generated using the cn.MOPs tool. This page displays the identified CNV region’s (gain or loss) of each soybean line in both searchable tabular and PDF format (Fig. 4f).

## Discussion

There are several challenges including data storage, data transfer, computing time, and availability of computing resources that accompany large genomic scale studies in biological organisms. Genomic variation studies on germplasm datasets in crops are no exceptions. Advances in high-performance computing and cloud storage technology can provide solutions for such challenges, but are generally out of reach for typical biological researchers. With PGen genomics variation analysis workflow development and availability, we have provided an efficient and easy-to-use analytics solution for biological users to address their needs for large-scale resequencing data analysis using HPC and cloud resources. For less computer savvy biological researchers, the web-based implementation in SoyKB allows them to still leverage the same scalable resources and solutions, but in an easy-to-use, non-tedious manner. The SoyKB NGS resequencing browser platform and online PGen workflow system allow users to easily submit analyses and access results via webpages. The workflow utilizes HPC resources from XSEDE and cloud storage from iDS to conduct NGS resequencing analysis and can be customized to work with other organisms as well. The workflow system is very flexible and additional local or remote computing resource can be easily incorporated. PGen can currently be run using three computing resources. First, we have the Pegasus resources of the Informatics Science Institute (ISI). The second resource comes from the Stampede and Wrangler high-performance computing cluster of TACC. The third resource is the XSEDE gateway allocation, which has been setup for SoyKB users utilizing the PGen online workflow. We are also building a fourth computing resource locally utilizing HPC resources at the University of Missouri-Columbia to provide PGen execution. More computing resources can be added as they become available to users in future.

PGen, together with its source code, is freely available to academic users via GitHub. It outlines best practices for efficient utilization of distinct and unique cyberinfrastructure (CI) resources available through multiple providers, with an emphasis on creating extensible and scalable workflows that can be easily modified and deployed. A similar approach can be utilized for designing many other bioinformatics analysis pipelines using the Pegasus workflow management system (Pegasus-WMS).

## Additional file

Additional file 1: PGen workflow user guide. This is a PGen user guide file for Linux users. In this file, users will be guided regarding applying for computational resources access and starting PGen workflows using provided sample datasets. Users can prepare their own datasets and run the PGen workflow with their own configurations. (DOCX 27 kb)
