# Supplementary material for: PGen: large-scale genomic variations analysis workflow and browser in SoyKB
Source: BMC Bioinformatics. 2016 Oct 6;17(Suppl 13):337. doi: 10.1186/s12859-016-1227-y (PMC5074001; doi:10.1186/s12859-016-1227-y)
Supplement: Additional file 1: — PGen workflow user guide. This is a PGen user guide file for Linux users. In this file, users will be guided regarding applying for computational resources access and starting PGen workflows using provided sample datasets. Users can prepare their own datasets and run the PGen workflow with their own configurations. (DOCX 27 kb) [file 12859_2016_1227_MOESM1_ESM.docx]

**I Account creation and request access to resources:**

1. **Create an iPlant account**

Create a user account at http://user.iplantcollaborative.org/ and request access to iPlant data store.

1. **Create a Pegasus account**

Pegasus system requires ssh key for login. Generate a public key in local linux system using the command as below and send the key to Mats Rynge ([rynge@isi.edu](mailto:rynge@isi.edu)) for requesting Pegasus permission.

**$ ssh-keygen -t rsa -b 2048**

**To run the workflow using TACC Stampede resource, step 3 and 4 are required. Else workflow can be run in distributed mode using step 1 and 2 only.**

1. **Create an XSEDE account**

Create a user account at <https://www.xsede.org/home> and submit a request for allocations <https://portal.xsede.org/allocations-overview>.

1. **Create a TACC account**

Once an XSEDE account was created, users will be assigned a TACC username via email and password can be changed by following instructions https://www.tacc.utexas.edu/.

**II Connect to workflow server:**

$ ssh username@workflow.isi.edu

**III** [**Running the NGS workflow**](https://pods.iplantcollaborative.org/wiki/display/ECS/Running+the+Soykb+workflow)**:**

1. **Download Pegasus workflow from Github:**

- The workflow is hosted on Github (https://github.com/pegasus-isi/PGen-GenomicVariations-Workflow). Log in and check out a version of the workflow in your home directory. (Note: your home directory will go away when you shut down the Atmosphere instance!) Clone the repository to get a local copy as below:

| **$ git clone https://github.com/pegasus-isi/PGen-GenomicVariations-Workflow.git** |
| --- |

- Note that the Git repository does not include the software required for the jobs. Grab the software tarball from http://www.isi.edu/~rynge/soybean/software.tar.gz and untar it in the top level workflow directory as below.

| **$ cd ~/PGen-GenomicVariations-Workflow**  **$ wget http://www.isi.edu/~rynge/soybean/software.tar.gz**  **$** **tar -xzf software.tar.gz** |
| --- |

- The workflow is controlled by a configuration file named ~/.pgen-workflow.conf
- Create the file with content as below:

| # **local refers to the submit host. Specify paths to a directory**  **# which can be used by the workflow as work space, and locations**  **# for local software installs.**  **[local]**  **work_dir = /local-scratch/%(username)s/pgen**  **irods_bin = /ccg/software/irods/3.2/bin**  **# tacc refers to configuration for the TACC Stampede**  **# supercomputer. To use this machine, you need an allocation**  **# (start with TG-) and you also need to know your username**  **# and storage group name for the system. The easiest way to**  **# obtain those is to log into the system, and run:**  **# cds; pwd**  **# This should return a path like: /scratch/00384/rynge. The**  **# storage group is the second level, and your username is**  **# last level.**  **[tacc]**  **allocation = your_xsede_allocation (example:TG-ABC1234)**  **username = your_tacc_username**  **storage_group = 00384** |
| --- |

1. **Inputs to workflow:**

Specifying input data is done in two files **inputs-fastq.txt** and **inputs-ref.txt**.

**a. inputs-fastq.txt**

**URLs are given in the irods://[resource]/[path] format.** **For example, to specify file** /iplant/home/shared/digbio/SoyKB/NGS_workflow/SRR1209394.fastq.gz

**to be preferably copied from the taccCorralRes resource, use:**

irods://taccCorralRes/iplant/home/shared/digbio/SoyKB/NGS_workflow/SRR1209394.fastq.gz

|  |
| --- |

Do not use comments or whitespace in the file. Make sure you have the permission of the data, you could check from the https://de.iplantcollaborative.org/de/

**b. inputs-refs.txt**

Reference genome should be in fasta format and it should be a zipped file. For example,

irods://taccCorralRes/iplant/home/shared/digbio/SoyKB/NGS_workflow/Zmays_284_AGPv3.gz

**c. *Single-end or pair-end fastq inputs**

Make sure to specify your input data type (pair or single), and change it in ‘main.conf’ file as below:

**$ cd ~/NGS-GenomicVariations-Workflow/conf/**

**$ cat main.conf**

**# single-end or pair-end**

**inputs-style = pair-end**

**d. *chromosomes.txt**

This input file saves the list of chromosomes for alignment and snp calling; user can make their own list according to the reference.

**zcat reference.fa.gz | grep “>” > chromosome.txt**

**e. *SNP Filtration Criteria:**

Default filtration for snps and indels:

**snp_filter = QD < 2.0 || FS > 60.0 || MQ < 40.0**

**indel_filter = QD < 2.0 || FS > 200.0 || MQ < 40**

Different filtration can be used and changed in ‘main.conf’ file by editing *‘snp_filter’* and *‘indel-filter‘* parameters.

1. **Outputs of workflow:**

Workflow generates bam, vcf and mergeGVCF output files. mergeGVCF can be used for future merge if needed. All outputs will be automatically transferred to iPlant data store during the workflow process named after workflow submission date.

For example if we have ‘SRR1209394.fastq.gz*’*as the input, workflow will generate below files as the outputs.

**SRR1209394 _addrepl.bai**

**SRR1209394 _addrepl.bam**

**SRR1209394 _indel_realigned.bai**

**SRR1209394_indel_realigned.bam**

**Submission_date-All.vcf**

**Submission_date-All.vcf.idx**

**Submission_date-All_filtered_indel.vcf**

**Submission_date-All_filtered_indel.vcf.idx**

**Submission_date-All_filtered_snp.vcf**

**Submission_date-All_filtered_snp.vcf.idx**

**Submission_date-mergeGVCF.vcf**

**Submission_date-mergeGVCF.vcf.idx**

1. **User Credentials:**

**The workflow requires 3 different user credentials:  a workflow ssh key to access data on the submit host, user’s iPlant password to access the data in iRods, and if user is running on a XSEDE resource, a X.509 grid proxy.**

Basic files are pulled from the submit host with scp. This is to keep the requirements on the submit host light, and make it easy to run the submit host as a cloud instance in for example Atmosphere. A workflow needs an ssh key:

| **$ mkdir -p ~/.ssh**  **$ ssh-keygen -t rsa -b 2048 -f ~/.ssh/workflow**  **(just hit enter when asked for a passphrase)**  **$ cat ~/.ssh/workflow.pub >>~/.ssh/authorized_keys** |
| --- |

1. **Access data from iPlant data store:**

To access data from the iPlant iRods repository, you need a file in your home directory named ~/irods.iplant.env, with 0600 permission and content as below:

**irodsHost data.iplantcollaborative.org**

**irodsPort 1247**

**irodsUserName YOUR_IRODS_USERNAME**

**irodsZone iplant**

**# Pegasus requirement**

**irodspassword ‘YOUR_IRODS_PASSWORD’**

To change permission running the command as below:

**# chmod 0600 irods.iplant.env**

1. **Running the workflow for execution in HTCondor pool:**

**./workflow-generator --exec-env distributed**

1. **Running the workflow on TACC Stampede or Wrangler:**

To run workflow on TACC, you need a X509 proxy. Create one with:

| **myproxy-logon -s myproxy.xsede.org -t 500:00 -l YOUR_XSEDE_USERNAME**  Then run as below:  **./workflow-generator --exec-env tacc-stampede**  **Or**  **./workflow-generator --exec-env tacc-wrangler** | |
| --- | --- |
|  | |

1. **Running and Monitoring of the workflow:**

Note that when Pegasus plans/submits a workflow, a work directory is created and presented in the output. This directory is the handle to the workflow instance and used by Pegasus command line tools. The first tool to use is pegasus-run, which will start the workflow:

| - ***pegasus-run [wfdir]*** |
| --- |

Some useful tools to know about:

- ***pegasus-status -v [wfdir]***

Provides status on a currently running workflow.

- ***pegasus-analyzer [wfdir]***

Provides debugging clues why a workflow failed. Run this after a workflow has failed.

- ***pegasus-statistics [wfdir]***

Provides statistics, such as walltimes, on a workflow after it has completed.

- ***pegasus-remove [wfdir]***

Removes a workflow from the system.

**Inputs for testing:**

***Inputs-fastq.txt:*** irods:///iplant/home/revieweraccess/PGen-workflow_data/SRR1209394.fastq.gz

***chromosome.txt:*** irods:///iplant/home/revieweraccess/PGen-workflow_data/chromosome.txt

***Inputs-ref.txt:*** irods:///iplant/home/revieweraccess/PGen-workflow_data/Zmays_284_AGPv3.gz

**9. A demo account for web-based PGen (**http://soykb.org/Pegasus/index.php**)**

Soykb account: pgendemo

Password: pgendemo
